# During high salt treatment myeloid p38α/MAPK fosters osteoclast activity and inflammatory macrophage responses promoting orthodontic tooth movement

**DOI:** 10.3389/fimmu.2025.1571268

**Published:** 2025-04-15

**Authors:** Agnes Schröder, Florian Fischer, Beatrice Reinert, Jonathan Jantsch, Peter Proff, Eva Paddenberg-Schubert, Christian Kirschneck

**Affiliations:** ^1^ Department of Orthodontics, University Hospital Regensburg, Regensburg, Germany; ^2^ Institute for Medical Microbiology and Hygiene, University Regensburg, Regensburg, Germany; ^3^ Institute for Medical Microbiology, Immunology and Hygiene, University Hospital Cologne and Faculty of Medicine, University of Cologne, Cologne, Germany; ^4^ Department of Orthodontics, University Hospital Bonn, Bonn, Germany

**Keywords:** orthodontic tooth movement, p38α/MAPK, bone remodeling, high salt diet, myeloid cells

## Abstract

**Introduction:**

During orthodontic tooth movement, sterile inflammatory processes and alveolar bone resorption occur in the periodontal ligament, involving myeloid cells such as macrophages and osteoclasts. The myeloid p38α/MAPK (mitogen-activated protein kinase) not only regulates the inflammatory response of macrophages and osteoclast differentiation but also the activation of the osmoprotective transcription factor NFAT5 (nuclear factor of activated T cells 5) under high-salt conditions. Therefore, this study aims to investigate the relative role of myeloid p38α/MAPK in orthodontic tooth movement as a function of extracellular salt content.

**Material and methods:**

Macrophages and osteoclasts were differentiated from the bone marrow of mice lacking p38α/MAPK expression in myeloid cells (*p38α* ^Δmyel^) and controls for RNA analysis and calcium phosphate resorption assay. Controls and *p38α* ^Δmyel^ mice were fed a low or a high salt diet for a total of two weeks. One week after the start of the diet, an elastic band was inserted between the first and second molar to induce orthodontic tooth movement. Atomic absorption spectrometry was used to assess the sodium balance of the jaw bone tissue. RNA was isolated from the periodontium of the first molar, osteoclast numbers and extent of orthodontic tooth movement were assessed.

**Results:**

*Nfat5* mRNA was increased in macrophages and osteoclasts *in vitro* and in the periodontium *in vivo* after high salt treatment in control mice but not in *p38α* ^Δmyel^ mice. While there was no salt effect on interleukin-6 (*Il6*) gene expression, prostaglandin endoperoxide synthase-2 (*Ptgs2*) mRNA was upregulated in control but not in *p38α* ^Δmyel^ mice *in vitro* and *in vivo*. p38α/MAPK deletion increased osteoclast numbers after low and high salt diet. Of note, deletion of p38α/MAPK elevated osteoclast activity under control salt conditions but reduced osteoclast activity under high salt conditions. High-salt diet resulted in increased sodium ion deposition in the jaw of both genotypes, while tooth movement was only increased in control mice. In *p38α* ^Δmyel^ mice, high salt diet reduced the extent of orthodontic tooth movement, which could be explained by the reduced bone resorption of osteoclasts.

**Conclusion:**

We conclude that myeloid p38α/MAPK promotes macrophage *Ptgs2* expression and osteoclast activity in response to extracellular salt levels, thereby supporting orthodontic tooth movement.

## 1 Introduction

Orthodontic appliances are used to correct misaligned teeth through bone remodelling processes mediated by osteoblasts and osteoclasts (1–3). The mechanical force application during orthodontic tooth movement results in the infiltration of myeloid cells into the periodontal ligament (4).

Myeloid cells play a decisive regulatory role in orthodontic tooth movement. On the one hand, along with periodontal ligament fibroblasts, they mediate the sterile inflammation during orthodontic movement. On the other hand, they serve as precursor cells for bone-resorbing osteoclasts.

In the pressure zone osteoblasts and periodontal ligament fibroblasts secrete receptor activator of nuclear factor-κB (RANKL), macrophage colony-stimulating factor (M-CSF), and osteoprotegerin (OPG), which are largely responsible for the differentiation of osteoclast progenitor cells into active osteoclasts (3, 5). Osteoclasts differentiate from myeloid cells and facilitate bone resorption after force appliance in the pressure zones (5). The combination of bone formation and resorption results in orthodontic tooth movement.

Next to bone remodelling, a pseudoinflammatory tissue response is induced by orthodontic treatment, which is regulated by many complex signaling cascades, cell types, and exogenous factors. The p38α/MAPK protein, as a mitogen-activated protein kinase, is responsible for modulating both of these processes (6–8). Although four different p38 isoforms are known, only p38α/MAPK is expressed by osteoclasts (7). Moreover, p38α/MAPK is partly responsible for the regulation of tooth movement and bone remodeling (8–10). In addition, this signaling pathway has been the focus of clinical research, particularly in diseases such as osteoarthritis and osteoporosis (11). Activation of p38α/MAPK is generally mediated by a wide variety of stimuli such as proinflammatory mediators, heat shock, UV or X-ray radiation (6, 12). During orthodontic tooth movement myeloid p38α/MAPK played a regulatory role by modulating osteoclastogenesis (10).

Many chronic diseases today are due to Western diets defined by a high intake of saturated fat, trans fat, sugar, salt, and a lack of fruits and vegetables (13). This is particularly evident in salt intake studies: They suggest that humans consume an average of about 10 g of salt per day, and although sodium ions, the cations in table salt, are essential mineral constituents, 0.5 g per day are recommended to maintain vital functions (14–16). Compared to this, the amount ingested appears to be high. This is also criticized by institutions such as the World Health Organization (14) and other bodies (13, 14, 16–18), which recommend an intake of 5 g per day.

Salt can affect orthodontic tooth movement as well. High salt diet enhances orthodontically induced tooth movement in mice depending on the osmoprotective transcription factor nuclear factor of activated T-cells 5 (NFAT5) (19) whose coupling with p38α/MAPK has already been demonstrated earlier (20, 21). High salt influences both osteoclastogenesis and bone resorbing activity of already differentiated osteoclasts. Elevated sodium chloride concentrations inhibit differentiation into osteoclasts while the bone resorption activity of already differentiated osteoclasts was increased (19, 22, 23).

The aim of this study was to investigate the relative contribution of p38α/MAPK to orthodontic tooth movement during high salt conditions. The influence of salt and p38α/MAPK on macrophages and osteoclasts during mechanical strain and their relative contribution to orthodontic tooth movement and bone remodelling processes will be analyzed in this work.

## 2 Material and methods

### 2.1 Cell culture approach

Bone marrow-derived macrophages were isolated from the tibia and femur of LysM^WT^ *p38α* ^fl/fl^ (control) and LysM^Cre^ *p38α* ^fl/fl^ (*p38α* ^Δmyel^) mice. *p38α* ^Δmyel^ mice do not express p38α/MAPK in myeloid cells such as macrophages and osteoclasts (24, 25). For generation of macrophages from the bone marrow, we followed a well-established protocol (26). Briefly, under sterile laminar air flow conditions, the bones were cut at the level of the proximal and distal epiphysis. The bone marrow was flushed out with 10 ml PBS (14190094, Life Technologies) and centrifuged for 5 min at 1400 rpm). To lyse the erythrocytes in the pellet, 5 ml of hemolysis buffer (0.4 g ammonium chloride (1.01145, Merck KGaA), 0.05 g potassium hydrogen carbonate (1.04854.0500, Merck), 0.01 ml 0.5 M EDTA pH8.0 (141696.0914, Applichem) in 50 ml H_2_O_dd_) were added. After 5 min at room temperature 10 ml PBS (14190094, Life Technologies) were added and samples were centrifuged for 5 min at 1400 rpm). The pellet was resuspended in 10 ml DMEM-high glucose (D5671, Sigma Aldrich) supplemented with 10% FBS (P30-3302, PAN), 1% antimycotica/antibacteria (A5955, Sigma Aldrich), 1% Glutamine (G7513, Sigma Aldrich) and 30 ng/ml M-CSF (macrophage colony-stimulating factor; 576404, Biolegend) and 10^6^ cells were seeded per dish and incubated for seven days.

For experiments with macrophages, 200,000 cells per ml were seeded in RPMI medium (61870-010, Thermo Fisher Scientific) supplemented with 10% FBS and 1% antimycotica/antibacteria (A5955, Sigma Aldrich) containing different NaCl concentrations. The Na^+^ concentration of RPMI medium (NaCl: -) was 140 mM. For high salt treatment (NaCl: +) 40 mM NaCl was added to RPMI medium resulting in a final concentration of 180 mM. Macrophages were cultured in different salt containing media for at least 24 h for the pressure experiments. Four hours before harvesting, pressure application (2 g/cm^2^) was performed using a zirconia plate (27).

For experiments with osteoclasts, 200,000 cells were seeded per ml in MEM-medium (F0925, Biochrom) with 30 ng/ml M-CSF and 50 ng/ml RANKL (receptor activator of NFkB ligand; 577102, Biolegend) and differentiated for another five days. Then medium was replaced with RPMI medium 61870-010, Thermo Fisher Scientific) supplemented with 10% FBS and 1% antimycotica/antibacteria (A5955, Sigma Aldrich) containing different NaCl concentrations (140 mM Na^+^; 180 mM Na^+^) for at least 24 h. Four hours before harvesting, pressure application (2 g/cm^2^) was performed (27).

### 2.2 Animal experimental approach

Animal experiments were performed in compliance with German laws (55.2.2-2532-2-567). LysM(Lyz2)^WT^ *p38α* ^fl/fl^ (control) and LysM(Lyz2)^Cre^ *p38α* ^fl/fl^ (*p38α* ^Δmyel^) were studied.

Eight-week-old male mice were fed either a low-salt diet (<0.03% sodium chloride (NaCl, ssniff) and tap water) or a high-salt diet (4% NaCl (ssniff) and 0.9% saline) for a total of two weeks. After one week on the specific diets, the animals were anesthetized and an elastic band (diameter 0.3 mm; Inwaria) was inserted between the first and second molar of the right upper jaw (28). After one week the animals were euthanized and the upper jaws were collected for µCT analysis and RNA isolation. For µCT, maxillary specimens from control and *p38α* ^Δmyel^ mice were fixed overnight in 5% formalin solution (10, 28, 29). For RNA analysis, tissues were placed in liquid nitrogen immediately after collection. These tissue samples were stored at -80°C until further use.

### 2.3 RNA analysis

Essentially, RNA analysis was performed as described earlier (19, 22, 30) The Purelink RNA Mini Kit (12183018A, Thermo Fisher Scientific) was used to isolate RNA from murine tissue according to the manufacturer´s instructions as described earlier (10, 29). For RNA isolation from cell culture samples 250 μl RNA Solv Reagent (R6830-01, VWR) were pipetted onto the cells after the pressure application (30, 31). This solution was transferred to a tube and 100 μl chloroform were added. After vortexing for 30 sec and incubation for 15 min on ice, the samples were centrifuged (15 min, 4°C, 13,000 rpm) and the upper, aqueous phase was transferred to a new tube containing 500 μl of ice-cold isopropanol (20,842,330, VWR). The samples were incubated overnight at -80°C. They were pelleted for 30 min at 4°C and 13,000 rpm. The pellet was washed twice with 500 μl 80% ethanol (32221, Sigma Aldrich). The pellet was dried and resuspended in 20 μl RNase-free H_2_Odd (L0015, Biochrom). Quantitative determination of total RNA was performed by photometric nanodrop measurement (N60, Implen). For murine tissue samples, equal concentrations of RNA were prepared in a final volume of 4 μl and mixed with 1 μl Luna Script RT SuperMix Kit (E3010L, NEB). Conversion to cDNA was performed in Thermocycler Tone 96G (Analytik Jena) by incubation for 2 min at 25°C, 10 min at 55°C, and 1 min at 95°C. For cell culture samples equal amounts of RNA were prepared in a final volume of 5.5 μl. The master mix was prepared from oligodT18 primer (SO132, Thermo Scientific), random hexamer primer (SO142, Thermo Scientific), 10 mM dNTPs (L785, Carl Roth), RNase inhibitor (EO0381, Thermo Scientific), M-MLV reverse transcriptase (M170B, Promega) and M-MLV buffer (M531A, Promega) and 4.5 µl per sample was added. Samples were incubated in the Tone 96 G thermal cycler (Analytik Jena) for 1 h at 37°C and 2 min at 95°C. The required primer mix consisted of 0.25 μl of each primer (**Table 1**), 5 μl Luna Universal qPCR mix (M3003E, BioLabs), and 3 μl RNase-free H_2_O_dd_ (L0015, Biochrom). For each sample, 1.5 μl cDNA and 8.5 μl primer mix were pipetted in duplicates into 96-well plates (712282, Biozym Scientific). These were sealed with foil (712350, Biozym Scientific) and briefly centrifuged. RT-qPCR analysis was performed in the Mastercycler realplex (Eppendorf). Samples were heated to 95°C for 10 min. This was followed by 45 cycles (10 sec 95°C, 20 sec 60°C, 8 sec 72°C). Data were analyzed using the 2^-ΔΔCT^ method with normalization to the reference gene *Eef1a1* (**Table 1**).

**Table 1:** Primers of the reference and target genes used for quantitative PCR (19).

Gene	Name	5´-*forward* primer-3´	5´-*reverse* primer-3´
*Eef1a1*	eukaryotic translation elongation factor 1-Alpha-1	AAAACATGATTACAGGCACATCCC	GCCCGTTCTTGGAGATACCAG
*Il6*	interleukin 6	TAGTCCTTCCTACCCCAATTTCC	TTGGTCCTTAGCCACTCCTTC
*Nfat5*	Nuclear factor of activated T cells 5	AAATGACCTGTAGTTCTCTGCTTC	GCTGTCGGTGACTGAGGTAG
*Ptgs2*	prostaglandin-endoperoxide synthase 2	TCCCTGAAGCCGTACACATC	TCCCCAAAGATAGCATCTGGAC

### 2.4 Western blot analysis

Immunoblotting was performed as described earlier (31). The proteins were isolated using 8 M urea (U5378, Sigma Aldrich) supplemented with protease inhibitor (10320015, Thermo Scientific). The lysates were incubated on ice for 15 min. After centrifugation (15 min, 4°C, 13,000 rpm) the pellet was discarded. The protein concentration in the supernatant was determined using a Bradford test (K015, Carl Roth). Equal protein concentrations were mixed with 6x sample buffer (3.75 ml 1 M Tris/HCl, pH6.8 (T1503, Sigma-Aldrich); 3 ml glycerol (3908.1, Carl Roth); 1.2 g SDS (8029.1 Carl Roth); 0.06 g bromphenolblue (T116.1, Carl Roth) and 14 14 mg/ml DTT (6908, Carl Roth) and heated for 7 min at 70°C, homogenized on ice and centrifuged (7 min, 4°C, 7,000 rpm). Proteins were separated on a 8% polyacrylamide gels and transferred to a PVDF membrane (T830, Carl Roth). After blocking the membranes in 5% milk in Tris buffered saline with 1% Tween for one hour at room temperature, the membranes were incubated overnight with a NFAT5-antibody (1:1,000; PA1-023, Invitrogen) and ACTIN (1:3,000; CA2066, Sigma-Aldrich). After incubation in the primary antibody, the membranes were washed three times with TBS-T for 10 min. The membranes were then incubated for 1 h with an HRP-conjugated secondary antibody (611-1302, Rockland), which was diluted 1:5,000 in 5% milk in TBS-T. Before developing, the membranes were washed again in TBS-T as described above. The software GenoCapture Version 7.01.06 (VWR) was used to visualize the protein bands. For this purpose, the membranes were coated with Immobilon Forte Western HRP Substrate (WBLUF0100, Merck Millipore). Immobilion Crescendo Western HRP Substrate (WBLUR0100, Merck Millipore) was used for the detection of ACTIN.

### 2.5 Enzyme-linked immunosorbent assays

ELISAs were performed as described earlier (30). To prepare the ELISAs, the supernatants stored at -80°C were thawed at least 30 min before the start of the experiment. Each ELISA was performed according to the manufacturer’s instructions and with a kit specifically for the antibody. Prostaglandin E2 (MBS266212, MyBioSource) and Interleukin-6 (MBS335514, MyBioSource) were analyzed.

### 2.6 Calcium phosphate resorption assay

CaP resorption assays were performed as described earlier (19, 22). Under sterile conditions, 6-well plates (353046, Omnilab) were coated with calcium phosphate. For this purpose, 50 mM Tris buffer (T1503, Sigma Aldrich) was adjusted to pH7.4 using HCl (X942, Carl Roth). In addition, a calcium stock solution was prepared composed of 25 mM calcium chloride monohydrate, 1.37 M NaCl (3957, Carl Roth), 15 mM magnesium chloride hexahydrate and Tris buffer pH7.4. The phosphate stock solution was prepared from 11.1 mM Na_2_HPO_4_∙H_2_O (K300, Carl Roth), 42 mM sodium hydrogen carbonate and Tris buffer pH7.4. All solutions were sterile filtered and proportionately mix: 50% Tris buffer pH7.4, 25% calcium phosphate and 25% phosphate stock solution. After adding 1.2 ml of this solution per well, the cell culture plates were incubated at room temperature for three days. The solution was aspirated and replaced with 1.2 ml of calcium phosphate (CaP) solution per well. CaP solution was freshly prepared by adding 41 ml HCl to 0.8 l H_2_O_dd_. In addition, 2.25 mM Na_2_HPO_4_-H_2_O, 0.14 M NaCl, 50 mM Tris were added. The solution was sterile-filtered before use. After adjusting pH7.4 with HCl, 4 mM CaCl_2_-H_2_O was added and made up to 1l with H_2_O_dd_. After incubation at room temperature for one day, CaP solution was removed, and 70% ethanol (32221, Sigma Aldrich) was briefly pipetted on for sterilization. The coated wells were cleaned twice with Ultra Pure Water (L0015, Biochrom) and dried overnight at 37°C. Before applying the cells, FBS (P30-3302, PAN) was added to the wells and incubated for 1 h at 37°C. After performing the experiments, the coated CaP cell culture plates were washed twice with PBS (14190094, Life Technologies). Cells were removed with 1 M NaCl (3957, Carl Roth) in 0.2% Triton-X-100 (T9284, Sigma Aldrich) for 10 min and then washed with Ultra Pure Water. After 400 μl silver nitrate solution (AgNO_3_; 7908, Carl Roth) was added for staining, cell culture plates were exposed to UV lamp for 45 min at room temperature. Subsequently, the wells were washed by adding distilled water and microscopic images were obtained. The evaluation was performed by ImageJ.

### 2.7 Determination of sodium and potassium

Atomic absorption spectrometry (AAS) was used to determine the sodium content in the jaw with associated mucosa, essentially as described earlier (32, 33). The jaws of untreated control mice and orthodontically treated control and *p38α* ^Δmyel^ mice were dried at 70°C for 72 h to determine water content, incinerated at 550°C and resuspended in 5% nitric acid (X898.2, Carl Roth). The samples were diluted with 0.5% cesium chloride (1.02039, Sigma Aldrich) and mixed by inverting. The diluted samples were analyzed using the Thermo Scientific iCE 3000 Series AA Spectrometer (Thermo Scientific). The measured Na^+^+K^+^ content was divided by the relative water content of the tissue to calculate the concentration.

### 2.8 Evaluation of µCT images

The computed tomographic (CT) images were acquired with Phoenix vltomelxs 240/180 (GE Sensing & Inspection Technologies) and VG Studio Max 2022.2.0. software (Volume Graphics) was used to analyze the data sets at the OTH Regensburg as described earlier (10, 28, 29). Briefly, to create accurately evaluable and reproducible conditions, the sagittal, coronal, and horizontal planes of the jaw were aligned. The maxillae were positioned symmetrically and centrally in each plane. This resulted in a sagittal plane that ran lengthwise through the Sutura palatina mediana, a coronal plane whose dentition was at the same level on both sides, and a horizontal plane that corresponded to the masticatory plane. In all subsequent measurements, both the control side and the orthodontically treated side were recorded simultaneously. To obtain the proximal distance between the first and second molars the smallest distance at the level of the anatomical tooth equator was used as the measuring point.

### 2.9 TRAP staining

The osteoclasts were stained using tartrate resistant acid phosphatase (TRAP) staining, essentially as described earlier (10, 28). Sections were incubated overnight at 37°C in an incubator and then kept for two hours in xylene, which was also heated, for further deparaffinization and occasionally swirled. It was ensured that the paraffin ran off the vertically positioned slides. They were then placed twice in xylene at room temperature for 10 min and, after visual inspection, rehydration of the tissue was started. This was done by adding 100%, 96% and 70% denatured ethanol to the sections twice at five-minute intervals. Finally, they were rinsed with tap water and placed in distilled water. Sections were placed for 10 min in the pre-warmed TRAP buffer, which was freshly prepared using 3.28 g sodium acetate (6773.1, Carl Roth), 46 g di-sodium tartrate dihydrate (T110.1, Carl Roth) and 1 liter of double-distilled water, and adjusted to pH5 with hydrochloric acid (H/120/PB15, Fisher Chemical). This was followed by the TRAP staining solution for four hours at 37°C in the incubator, whereby the temperature promoted the activity of the enzyme and thus the staining. The staining solution was prepared from 40 mg Naphtol AS-MX Phosphate Disodium Salt (N5000, Sigma-Aldrich), 4 ml N-N-dimethylformamide (D4551, Sigma-Aldrich), 240 mg Fast Red Violet LB Salt (F3381, Sigma-Aldrich), 2 ml Triton X (T9284, Sigma-Aldrich) and 200 ml of the TRAP buffer. Finally, rinse in distilled water and immediately cover with Aquatex (1.08562.0050, Merck), taking care to remove air bubbles with tweezers and wipe off excess. Finally, the sections were dried overnight under the fume hood. To evaluate the osteoclast density of the alveolar bone all sections were digitized using the PANNORAMIC™ Digital Slide Scanner (3D Histech). The scans were analyzed with the Case Viewer software (version 2.2.1) and Image J (version 1.53e).

### 2.10 Statistics

Statistical analysis was performed using GraphPad Prism version 9.5 (GraphPad Software). The data were tested for normal distribution using the Shapiro Wilk test. For data without pressure application or OTM either an unpaired two-tailed T test or a Mann-Whitney U test was calculated, depending on the results of normality testing. For data of the pressure or OTM groups an ANOVA with Holm-Sidak’s *post hoc* test or a Welch-corrected ANOVA with Dunnett’s T3 *post hoc* test was performed, depending on normal distribution. In the graphs, each symbol corresponds to a data point. The horizontal lines represent the mean and the vertical lines the standard error.

## 3 Results

### 3.1 p38α/MAPK affects the expression of the osmoprotective transcription factor NFAT5 during mechanical strain and high salt treatment

First, we tested the relative contribution of high salt and p38α/MAPK on the Na^+^ and K^+^ concentration in the jaw with associated mucosa and the effects on *nuclear factor of activated T cells 5* (*Nfat5*) gene expression in the periodontal ligament.

**Figure 1:**
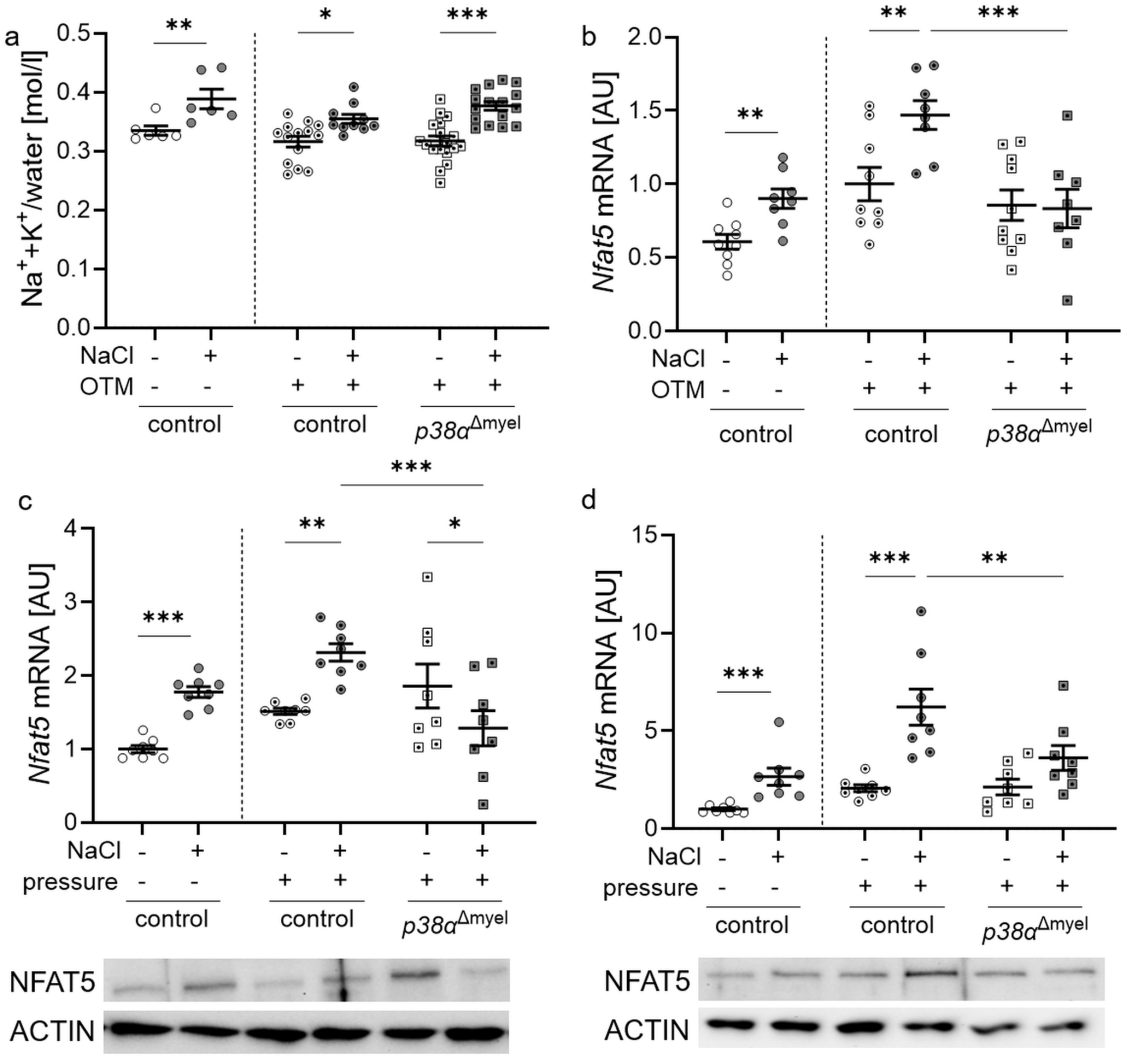
Evaluation of Na^+^+K^+^/water in the jaw with associated mucosa **(a)** and gene expression of *Nfat5* in control mice and mice lacking p38α/MAPK in myeloid cells (*p38α* ^Δmyel^) after two weeks on low or high salt (NaCl) diet **(b)**. *Nfat5* mRNA and protein expression was determined in bone marrow-derived macrophages **(c)** and osteoclasts **(d)**. Cells were either treated with NaCl (+) or left untreated (–) without and with compressive strain. Statistics: Data without OTM or pressure application: unpaired t test **(b, c)** or Mann-Whitney U Test **(a, d)**; Data with OTM or pressure application: ANOVA with Holm-Sidak’s *post hoc* test *P<0.05; **P<0.01; ***P<0.001.

In control mice without orthodontic treatment, high salt diet increased Na^+^+K^+^ concentrations in the lower jaw including the mucosa (*P*<0.008). The same was observed in orthodontically treated control mice (*P*=0.014) and mice lacking p38α/MAPK in myeloid cells (*p38α* ^Δmyel^, *P*<0.001; **Figure 1a**). Accordingly, gene expression of *Nfat5* was elevated in control mice without (*P*=0.001) and with OTM (*P*=0.005; **Figure 1b**). In *p38α* ^Δmyel^ mice, however, the high NaCl diet failed to increase *Nfat5* expression (*P*=0.869; **Figure 1b**).

To determine the relative role of macrophages and osteoclasts, both cell types were stimulated with normal (140 mM Na^+^) and high salt (180 mM Na^+^) in combination with mechanical strain, respectively, and the gene expression of *Nfat5* was analyzed. Under control conditions without mechanical strain (*P*<0.001) and after pressure application, bone marrow-derived macrophages from control mice elevated *Nfat5* mRNA (*P* = 0.005; **Figure 1c**) and protein levels after addition of NaCl (**Figure 1c**). This effect was abolished, when p38α/MAPK was missing (*P*<0.001) and *Nfat5* mRNA was significantly reduced with additional NaCl (*P*=0.041; **Figure 1c**).

After bone marrow-derived macrophages were differentiated into osteoclasts, NaCl was added to the medium. *Nfat5* mRNA (P<0.001) and protein was increased in osteoclasts from control mice under high salt conditions without and compressive strain (**Figure 1d**). This high salt effect was abolished, when p38α/MAPK was missing (*P*=0.003). This data demonstrate that p38α/MAPK is involved in the regulation of *Nfat5* gene expression in mice, macrophages and osteoclasts during mechanical strain.

### 3.2 p38α/MAPK impacts on Ptgs2 but not on Il6 gene expression during high salt and mechanical strain

To characterize the contribution of p38α/MAPK to the sterile inflammation occurring during orthodontic treatment, we analyzed the expression profiles of *Nfat5* target genes interleukin-6 (*Il6*) and prostaglandin endoperoxide synthase 2 (*Ptgs2*) in control and *p38α* ^Δmyel^ mice as well as in bone marrow-derived macrophages of both genotypes (34–36).

**Figure 2:**
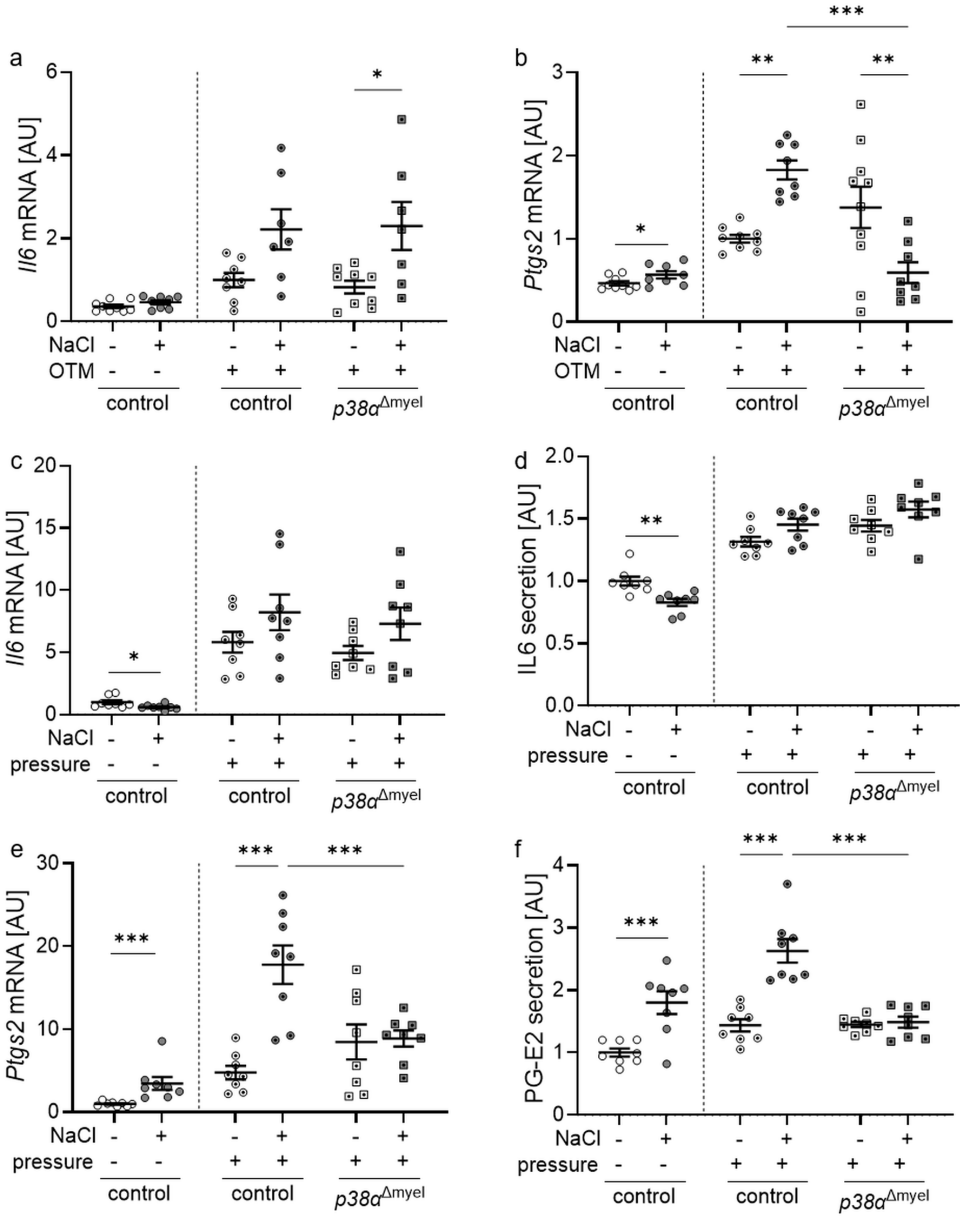
Gene expression of *Il6* **(a)** and *Ptgs2* **(b)** in the periodontal ligament of untreated control mice and orthodontically treated control mice and mice lacking p38α/MAPK in myeloid cells (*p38α* ^Δmyel^) after two weeks on low or high salt (NaCl) diet. Gene and protein expression of *Il6* **(c, d)** and *Ptgs2* / PG-E2 **(e, f)** in bone marrow-derived macrophages treated with additional NaCl or left untreated without and with compressive strain. Secretion of IL6 **(e)** and production of PG-E2 **(f)** was determined in the cell culture supernatant of bone marrow-derived macrophages without or with additional NaCl exposed to compressive strain or left untreated. Statistics Data without OTM or pressure application: unpaired t test **(a, b, d, f)** or Mann-Whitney U Test **(c, e)**; Data with OTM or pressure application: ANOVA with Holm-Sidak’s *post hoc* test **(a-f)**. *P<0.05; **P<0.01; ***P<0.001.

Gene expression of *Il6* tended to be increased by high salt diets in controls, but upregulated in *p38α* ^Δmyel^ mice (*P*=0.045; **Figure 2a**). High NaCl diet increased gene expression of prostaglandin endoperoxide synthase 2 (*Ptgs2*) in control mice (*P*=0.004; **Figure 2b**). This effect was reversed, when p38α/MAPK was deleted in myeloid cells, leading to a significant reduction of *Ptgs2* mRNA compared to control mice (*P*<0.001; **Figure 2b**).

In bone marrow-derived macrophages of control mice, high salt conditions reduced gene expression (*P*=0.021; **Figure 2c**) and protein secretion (*P*=0.001; **Figure 2d**) of *IL6* under control conditions without compressive strain. After pressure application there was no significant effect of NaCl on *IL6* mRNA expression (*P*=0.231; **Figure 2c**) or secretion (*P*=0.234; **Figure 2d**) detectable. Without and with mechanical strain, gene expression of *Ptgs2* was elevated with addition of NaCl (*P*<0.001; **Figure 2e**). Deletion of p38a/MAPK inhibited this NaCl effect (*P*<0.001) in analogy to *Nfat5* mRNA expression. The same was detected at the protein level investigating prostaglandin-E2 (PG-E2) secretion (**Figure 2f**). This data show a contribution for p38α/MAPK in the high salt induced regulation of the *Nfat5* target gene *Ptgs2*, but not *Il6*, during mechanical strain in mice and in macrophages *in vitro*.

### 3.3 Impact of myeloid p38α/MAPK during high salt treatment and compressive strain in osteoclasts derived from bone marrow

**Figure 3:**
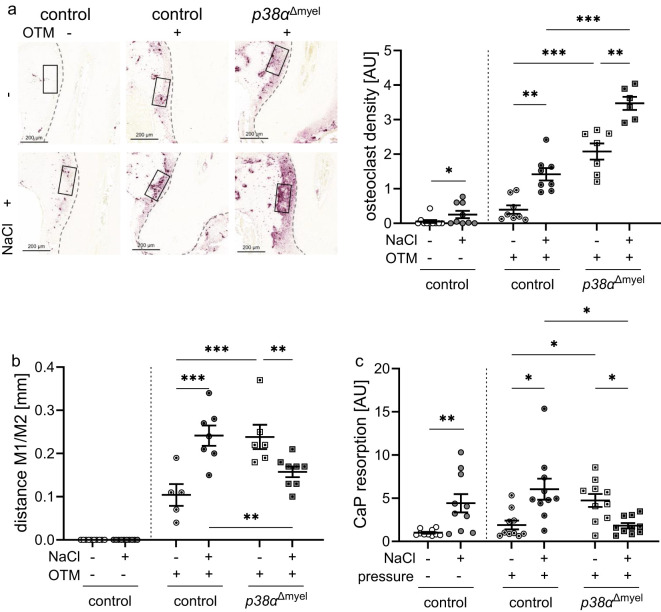
Osteoclast density determined in the indicated region of interest [black box; **(a)**] and distance between the 1^st^ (M1) and 2^nd^ (M2) molar **(b)** of untreated control mice and orthodontically treated control mice and mice lacking p38α/MAPK in myeloid cells (*p38α* ^Δmyel^) after two weeks on low or high salt (NaCl) diet. CaP resportion assay of bone marrow derived osteoclasts **(c)** treated with NaCl or left untreated without and with compressive strain after differentiation. Statistics: Data without OTM or pressure application: Mann-Whitney U Test **(a, c)**; Data with OTM or pressure application: ANOVA with Holm-Sidak’s *post hoc* test **(b)** or Welch-corrected ANOVA with Dunnett´s *post hoc* test **(a, c)**. *P<0.05; **P<0.01; ***P<0.001.

Next to macrophages, myeloid cells can differentiate to osteoclasts and promote alveolar bone resorption during orthodontic tooth movement. On a high-salt diet, the number of osteoclasts increased in control mice on the control side of the lower jaw (*P*=0.026). The same was observed in the orthodontically treated control side (*P*=0.002) and in *p38α* ^Δmyel^ mice (*P*=0.003; **Figure 3a**). Mice without p38α/MAPK expression in myeloid cells displayed more osteoclasts under low and high salt diet (*P*<0.001). As expected there was no distance between the 1^st^ and the 2^nd^ molar on the control side of the lower jaw (**Figure 3b**). In line with osteoclasts numbers and earlier publications (19), high salt increased the extent of orthodontic tooth movement in control mice (*P*=0.001; **Figure 3b**). In view of the further increase in osteoclast numbers (*P*=0.002) in the p38α/MAPK-deficient situation, it could be hypothesized that this would result in increased tooth movement under high-salt conditions. With high salt, however, tooth movement in *p38α* ^Δmyel^ mice was significantly reduced compared to the control littermates or *p38α* ^Δmyel^ mice fed a low salt diet (*P*=0.016; **Figure 3b**). This suggested that the resorptive capacity of the osteoclasts in *p38α* ^Δmyel^ mice might be compromised. To test resorption activity of the osteoclast derived from the bone marrow of control and *p38α* ^Δmyel^ mice, CaP resorption assays were performed. After high salt treatment of osteoclasts an increased CaP resorption was observed without (P=0.002) and with pressure (P=0.032; **Figure 3c**). When p38α/MAPK was missing osteoclast activity was increased under normal salt conditions resulting in a higher CaP resorption (*P*=0.025). Under high salt conditions, however, osteoclast activity and CaP resorption were reduced in osteoclasts derived from *p38α* ^Δmyel^ mice (*P*=0.027; **Figure 3c**). From this data, we conclude that while osteoclast differentiation was not affected by p38α/MAPK during high salt exposure, their resorption activity depended on p38α/MAPK, ultimately affecting the extent of orthodontic tooth movement during high salt treatment.

## 4 Discussion

High-salt diet increased Na^+^+K^+^/water content in the mandible with associated oral mucosa in both genotypes. However, increased gene expression of the osmoprotective transcription factor *Nfat5* could only be detected in the periodontal ligament, macrophages and osteoclasts of the control animals and not in *p38α* ^Δmyel^ mice. These findings are in line with earlier publications, who reported a dependence of NFAT5 expression on the p38α/MAPK under high salt conditions (21, 37, 38).

Orthodontic tooth movement creates pressure and tension zones in the periodontal ligament. Bone regeneration processes take place at the tension zones, while at the pressure zones the alveolar bone is resorbed by osteoclasts (39, 40). During orthodontic tooth movement cytokines and prostaglandins are released after pressure application, leading to a sterile low-grade inflammatory response (3, 41, 42). These inflammatory mediators include interleukin-6 (IL6) and prostaglandin endoperoxide synthase-2 (PTGS2) (43) thereby modulating the differentiation and activation of osteoclasts (2, 44). High salt diet elevated the gene expression of *Ptgs2* in the periodontal ligament and in macrophages of control animals, while it was downregulated in *p38α* ^Δmyel^ mice. *Ptgs2* has already been reported to be increased by high salt conditions in macrophages (31) and its expression is among others regulated by NFAT5 (45, 46). Thus, the lack of induction of *Ptgs2* mRNA in *p38α* ^Δmyel^ mice can be attributed to a missing induction of NFAT5.

Under a high-salt diet, there was increased tooth movement in control mice. In a previous study, this high-salt effect could be attributed to increased osteoclast activity (19). Under high salt conditions, orthodontic tooth movement was reduced in *p38α* ^Δmyel^ mice. This data are in line with Kirschneck et al., who reported attenuated tooth movement in mice lacking p38α/MAPK in myeloid cells (10). The reduced resorption activity of p38/MAPK deleted osteoclasts under high salt could be an explanation for the decreased tooth movement under high salt conditions in *p38α* ^Δmyel^ mice. Cong et al. already demonstrated that p38α/MAPK could positively or negatively regulate osteoclast differentiation in a cell density- and age-dependent manner thereby affecting bone remodeling (8). The high salt phenotype of *p38α* ^Δmyel^ mice resembled the observed phenotype under normal salt diet (10). Here, decreased tooth movement and increased bone density were reported in *p38α* ^Δmyel^ mice. It has been shown that p38α/MAPK and dietary saline intake influence orthodontic tooth movement by affecting osteoclast activity. One possible mechanism by which p38α/MAPK affects osteoclasts that needs to be further investigated is through autophagy. In line with this, p38α/MAPK can block autophagy (47) and inhibition of autophagy is involved in osteoclast differentiation and activation (48). Moreover, high salt affects autophagy as well (49). These findings suggest that there might be an interplay between high salt exposure, p38/MAPK, autophagy and osteoclast activation and function.

Additional experiments are needed to elucidate the mechanistic underpinnings of this interaction. In addition to the fact that the mechanism of impaired osteoclast activity has not yet been fully elucidated, our research is limited by the fact that we performed both the gene expression analyses and the electrolyte measurements at the total tissue level of the jaw and surrounding mucosa, which, in addition to myeloid cells, also consists of periodontal ligament fibroblasts and other immune cells.

From a clinical point of view, the observed effects could potentially be used in orthodontic treatment, however a study in a mouse model can only give a bold assessment to the situation in humans upon orthodontic treatment. For example, the reducing osteoclast activity by locally inhibiting p38α/MAPK activity in osteoclasts might be useful in orthodontic patients with a history of periodontitis and high salt diet. In such cases, the specific inhibition of p38α/MAPK in osteoclasts might reduce the risk for an abnormal bone resorption.

Therefore, we conclude that myeloid p38 α/MAPK influences the expression of *Ptgs2* from macrophages and the osteoclast activity in dependence of the extracellular salt content thereby affecting orthodontic tooth movement.

## Data Availability

The original contributions presented in the study are included in the article/supplementary material. Further inquiries can be directed to the corresponding author.
